# The Role of Reductive Stress in the Pathogenesis of Endocrine-Related Metabolic Diseases and Cancer

**DOI:** 10.3390/ijms26051910

**Published:** 2025-02-23

**Authors:** Mitko Mladenov, Iliyana Sazdova, Nikola Hadzi-Petrushev, Rossitza Konakchieva, Hristo Gagov

**Affiliations:** 1 Institute of Biology, Faculty of Natural Sciences and Mathematics, Ss. Cyril and Methodius University, 1000 Skopje, North Macedonia; mitkom@pmf.ukim.mk (M.M.); nikola@pmf.ukim.mk (N.H.-P.); 2 Department of Fundamental and Applied Physiology, Russian States Medical University, 117997 Moscow, Russia; 3 Department of Animal and Human Physiology, Faculty of Biology, Sofia University “St. Kliment Ohridski”, 1164 Sofia, Bulgaria; i.sazdova@biofac.uni-sofia.bg; 4 Department of Cell and Developmental Biology, Faculty of Biology, Sofia University “St. Kliment Ohridski”, 1164 Sofia, Bulgaria; r.konakchieva@biofac.uni-sofia.bg

**Keywords:** reductive stress, hormones, oxidative stress, metabolism, diabetes, cardiovascular disease, cancer, redox homeostasis

## Abstract

Reductive stress (RS), characterized by excessive accumulation of reducing equivalents such as NADH and NADPH, is emerging as a key factor in metabolic disorders and cancer. While oxidative stress (OS) has been widely studied, RS and its complex interplay with endocrine regulation remain less understood. This review explores molecular circuits of bidirectional crosstalk between metabolic hormones and RS, focusing on their role in diabetes, obesity, cardiovascular diseases, and cancer. RS disrupts insulin secretion and signaling, exacerbates metabolic inflammation, and contributes to adipose tissue dysfunction, ultimately promoting insulin resistance. In cardiovascular diseases, RS alters vascular smooth muscle cell function and myocardial metabolism, influencing ischemia-reperfusion injury outcomes. In cancer, RS plays a dual role: it enhances tumor survival by buffering OS and promoting metabolic reprogramming, yet excessive RS can trigger proteotoxicity and mitochondrial dysfunction, leading to apoptosis. Recent studies have identified RS-targeting strategies, including redox-modulating therapies, nanomedicine, and drug repurposing, offering potential for novel treatments. However, challenges remain, particularly in distinguishing physiological RS from pathological conditions and in overcoming therapy-induced resistance. Future research should focus on developing selective RS biomarkers, optimizing therapeutic interventions, and exploring the role of RS in immune and endocrine regulation.

## 1. Introduction

Endocrine cells form endocrine glands or are dispersed in non-endocrine organs and release hormones into circulation. Hormones are signaling molecules with a diverse chemical nature, among others steroids (gonadal and adrenocortical hormones, and calcitriol), amino acid derivatives (e.g., epinephrine, norepinephrine, and dopamine), peptides (e.g., oxytocin, vasopressin, and angiotensin II), proteins (e.g., prolactin, insulin, and growth hormone), and glycoproteins (e.g., follicle-stimulating hormone (FSH), luteinizing hormone (LH), and thyroid-stimulating hormone (TSH; thyrotropin)). Transported via the bloodstream, they exert signaling effects on target cells at a distance from the site of release and regulate virtually all biological processes and functions through specific membrane, cytoplasmic, and/or nuclear receptor-activated intracellular signaling in target cells [1,2].

Cells, organs (e.g., the liver), and whole-body metabolism are under continuous endocrine control via hormonal secretion. Hormones regulate cell growth, proliferation, maturation, and death [2], as well as the cellular redox state [3]. The latter is defined as the balance between pro-oxidants and antioxidants, which maintain cellular redox homeostasis [4,5]. Cellular pro-oxidants include superoxide anion, hydrogen peroxide (H_2_O_2_), and other reactive oxygen species (ROS), as well as nitric oxide (NO) and its derivatives, known as reactive nitrogen species (RNS). Transitional metals such as iron and copper also contribute to redox regulation [5,6].

Several redox couples—reduced glutathione/oxidized glutathione (GSH/GSSG), reduced nicotinamide adenine dinucleotide phosphate/oxidized nicotinamide adenine dinucleotide phosphate (NADPH/NADP^+^), reduced nicotinamide adenine dinucleotide /oxidized nicotinamide adenine dinucleotide (NADH/NAD^+^), and thioredoxins (TrxSH/TrxSS)—act as redox buffers, working alongside essential redox enzymes such as glutathione reductase (GR), catalase (CAT), superoxide dismutases (SOD; SOD1-3), glutathione peroxidases (GPx; GPx1-8), glutaredoxins (GRX; GRX1-2), thioredoxin reductases (TRs), peroxiredoxins 1-6, and sulfiredoxin 1. These molecules play a crucial role in maintaining cellular redox homeostasis [7,8].

A significant deviation from the optimal redox state leads to oxidative stress (OS), characterized by an excess of pro-oxidants such as ROS and RNS, as well as oxidizing agents like GSSG, NAD(P)^+^, and TrxSS. In contrast, reductive stress (RS) occurs when the reducing capacity is disproportionately elevated [5,8,9]. RS is defined by the excessive accumulation of reducing equivalents, such as NADH and NADPH, leading to a pathological disruption of redox homeostasis. This imbalance arises from hyperactive metabolic pathways, including glycolysis and the pentose phosphate pathway (PPP), which result in an overproduction of antioxidants like GSH and Trx [5]. Similar to OS, RS is harmful to cellular physiology through multiple mechanisms, including impaired intracellular signaling and gene expression, the induction of OS, chronic inflammation, metabolic disorders, and certain cancers [10,11,12,13,14,15].

This review summarizes current data on the effects of RS and their interaction with OS in altering hormonal synthesis, secretion, and signaling. The objective is to shed light on common origin hormone-dependent disorders and pathological conditions arising from RS-induced disruptions in endocrine regulation and cancer progression.

## 2. RS as Regulator of Cellular Functions

### 2.1. Mechanisms of RS Generation and Signaling

Superoxide anion (O_2_^−^), produced by the electron transport chain (ETC), is converted into the more stable H_2_O_2_ in mitochondria. However, H_2_O_2_ can be further converted into the highly reactive and harmful hydroxyl radical (OH•) through the Fenton reaction. H_2_O_2_ is detoxified into water by GSH, with the concomitant formation of GSSG. The GSSG is then recycled back to GSH by GR, which utilizes NADPH, primarily derived from the PPP, as an electron donor.

**Figure 1:**
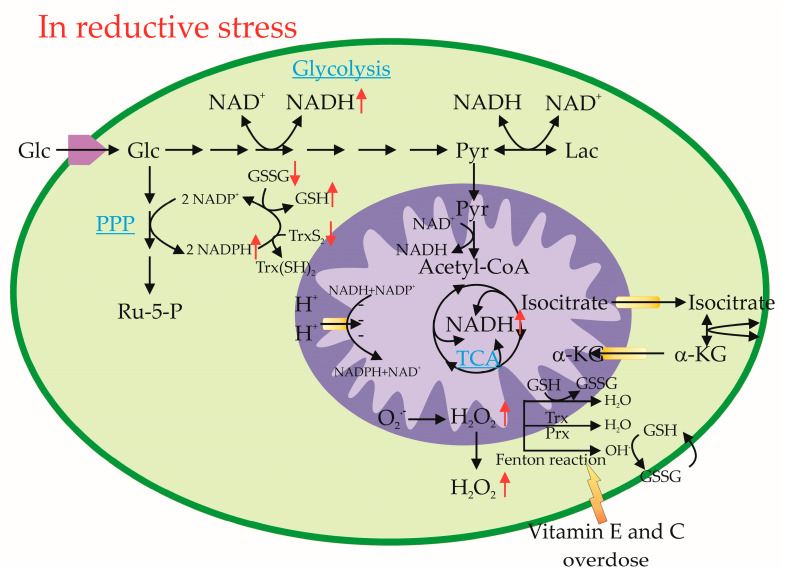
Metabolic pathways of ROS and reductive agents’ generation. Glucose in the cell can be metabolized in glycolysis or pentose phosphate pathway depending on the redox status of the cell. (α-KG: alpha-ketoglutarate; Glc: glucose; GSH: glutathione, reduced form; GSSG: oxidized glutathione; Lac: lactate; NAD(P)H: nicotinamide adenine dinucleotide (phosphate), reduced form; NAD(P)+: nicotinamide adenine dinucleotide (phosphate), oxidized form; PPP: pentose phosphate pathway; Pyr: pyruvate; TCA cycle: tricarboxylic acid cycle; Trx: thioredoxins). Black solid arrows (→) indicate the flow of metabolic pathways, showing the conversion of one molecule into another. Black double arrows (↔) represent reversible biochemical reactions. Red upward arrows (↑) indicate an increase in certain metabolites or stress markers. Red downward arrows (↓) indicate a decrease in metabolites or molecules of redox homeostasis. Curved arrows represent the cycling of redox molecules or electron transfer. The orange arrow labelled “Vitamin E and C overdose” indicates that excessive antioxidant supplementation may interfere with redox homeostasis, contributing to reductive stress.

The intracellular GSH level depends on cysteine availability, which is produced through the methionine cycle and transsulfuration pathway involving two ATP-dependent enzymatic steps—gamma-L-glutamyl-L-cysteine:glycine ligase followed by glutathione synthetase. Both pathways require essential cofactors such as folate, vitamins B_2_, B₆, and B₁_2_, methionine, and cysteine. Excessive multivitamin supplementation can drive the one-carbon metabolism toward excessive GSH production. Increased NADPH consumption due to elevated GSH/GSSG turnover stimulates the PPP to generate more NADPH (Figure 1).

On the other hand, increased GSH levels in dysfunctional mitochondria impair NADH oxidation, which ultimately contributes to RS. Mitochondrial complex I is the primary enzyme responsible for NADH metabolism and serves as a major electron donor for ATP synthesis [16]. GSH accumulation, genetic mutations, hypoxia, and decreased ATP demand can inhibit the ETC, leading to an increased NADH/NAD⁺ ratio and subsequent ROS generation [17,18]. Additionally, a deficiency of oxidized reductants such as GSSG and Trx can cause electron leakage, further promoting H_2_O_2_ formation [19].

In RS, an elevated NADH/NAD⁺ ratio leads to the excessive reduction of O_2_ to O_2_^−^, followed by the overproduction of H_2_O_2_. Increased TCA cycle activity or defective mitochondrial complexes contribute to NADH accumulation, which, in turn, exacerbates ROS production. Elevated ROS levels can shift energy metabolism from glycolysis to the PPP, increasing NADPH production as a countermeasure against OS. However, RS is worsened by the simultaneous accumulation of reducing equivalents (NADH, NADPH, GSH, TrxSH) and a lack of oxidized reductants (GSSG, TrxSS) as electron acceptors, leading to H_2_O_2_ leakage from mitochondria (Figure 1) [16].

**Figure 2:**
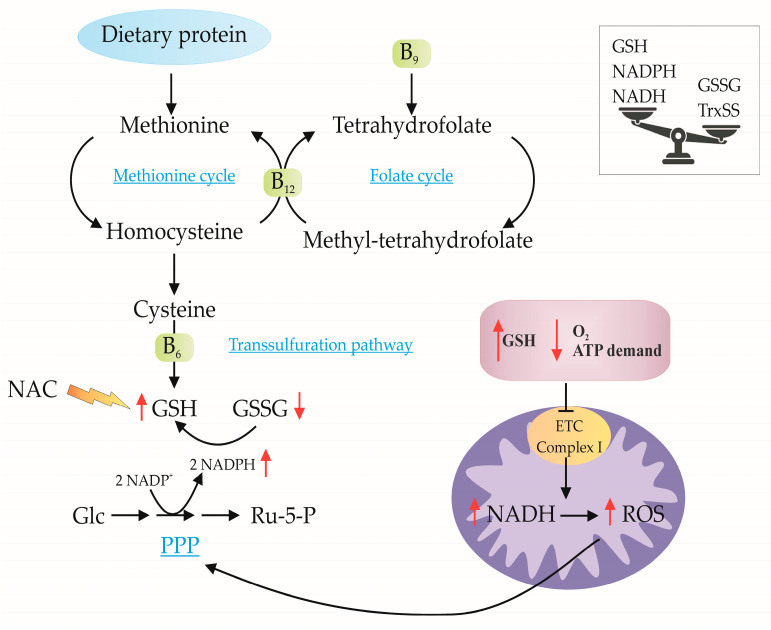
‘Antioxidant paradox’. Increased concentrations of reductive agents (GSH, NADH, NADPH) induce ROS generation but under worse environmental conditions. The antioxidant system of the cell is disarmed because the concentration of oxidized forms is too low. A high protein diet, high vitamin intake, and/or supplementation disturbs the redox state in the cell (ETC: electron transport chain; Glc: glucose; GSH: glutathione, reduced form; GSSG: oxidized glutathione; NAC: N-acetylcysteine; NAD(P)H: nicotinamide adenine dinucleotide (phosphate), reduced form; NAD(P)+: nicotinamide adenine dinucleotide (phosphate), oxidized form; PPP: pentose phosphate pathway; ROS: reactive oxygen species). Black solid arrows (→) represent direct biochemical conversions or metabolic pathways. Curved arrows (↻) indicate cyclic metabolic processes. Red upwardarrows (↑) indicate an increase in certain molecules or metabolic activities. Red downward arrows (↓) indicate a decrease in certain molecules or metabolic processes. Orange lightning bolt (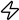) represents an external intervention or stimulus. Balancing scale symbol denotes the change of balance between oxidized and reduced forms of key molecules.

RS can paradoxically lead to an overproduction of ROS despite an abundant pool of antioxidants. This occurs when the reduced NADH/NAD⁺ pool exceeds the capacity of antioxidant defenses, even in the presence of an elevated GSH/GSSG and NADPH/NADP⁺ ratio. The excessive ROS production outpaces ROS scavenging potential, primarily due to a deficiency in oxidized reductants (mainly GSSG and Trx), which are required for the optimal function of enzymatic antioxidants [20,21]. The paradoxical increase in ROS, despite high antioxidant levels, was described by Halliwell in 2000 as the “antioxidant paradox” (Figure 2) [22].

The GSH/GSSG ratio and redox potential vary across different cellular compartments:

- In the cytosol, the ratio is ≥30:1–100:1, with a redox potential of −290 mV;
- In mitochondria, the ratio is >100:1, with a redox potential of ≤−300 mV;
- In the endoplasmic reticulum (ER), the ratio is 1:1 to 3:1, with a redox potential from −175 to −185 mV [23].

The relatively oxidizing environment of the ER is essential for disulfide bond formation and proper protein folding [24]. The primary intracellular GSH pool is located in the cytosol. However, cytosolic GSH can be transported into other compartments, particularly mitochondria, via three key transporters:

1. α-Ketoglutarate carrier;
2. Dicarboxylate carrier;
3. Tricarboxylate carrier [5].

### 2.2. Pro-Oxidant Effects of Antioxidants

Antioxidants are well-known ROS scavengers, protecting proteins, lipids, and DNA from oxidative damage and peroxidation. However, excessive doses of antioxidants can lead to cellular dysfunction by shifting the redox balance toward a reductive state, a condition known as RS [25,26]. This explains why antioxidants are not entirely effective in treating neurodegenerative diseases, chronic inflammation, cardiovascular diseases, and cancer. In some cases, they can even worsen disease progression by interfering with other therapies and reducing their effectiveness [27].

Tocopherol (vitamin E) has been shown to exhibit pro-oxidant activity under certain conditions. Through the Fenton reaction, it contributes to bone alterations and increases the risk of lung cancer (Figure 1) [28,29]. Similarly, β-carotene, a lipophilic antioxidant, is converted into vitamin A (retinol) in the small intestine and subsequently stored in the liver as a retinol ester [30]. However, at high doses, vitamin A exerts pro-oxidant effects, stimulating the synthesis of pro-inflammatory mediators such as tumor necrosis factor-alpha (TNF-α) and interleukin-8 [13,31].

Excessive retinol supplementation has been linked to an increased risk of lung cancer [32] and colorectal polyp formation [33]. These findings highlight the paradoxical effects of lipophilic antioxidants, which can shift from protective to harmful pro-oxidant activity depending on dosage and metabolic conditions.

The pro-oxidant effects of vitamin C (ascorbic acid) at high doses have been well-documented. Excessive vitamin C intake can lead to the accumulation of calcium oxalate crystals in the kidneys, as ascorbic acid is metabolized to oxalate, increasing the risk of kidney stone formation [34,35]. Additionally, vitamin C acts as a pro-oxidant agent by stimulating lipid peroxidation, which leads to cell damage. It also participates in peroxidative reactions involving iron and copper via the Fenton reaction, further contributing to OS [35]. Supplementing with high doses of vitamin C (500 mg/day) has been found to induce oxidative DNA damage in lymphocytes, emphasizing its potential cytotoxic effects (Figure 1) [36,37].

Chronic treatment with N-acetylcysteine (NAC), a precursor for GSH synthesis, has been shown to increase the NADH/NAD⁺ ratio, leading to RS. Prolonged NAC treatment resulted in mitochondrial oxidation and cytotoxicity in cardiomyocytes, demonstrating its potentially harmful effects on cardiac function [38]. NAC, in combination with vitamin E, has been reported to promote lung cancer progression [39]. The reduction in ROS levels caused by NAC disrupted p53 activity, leading to increased tumor cell proliferation and accelerated tumor growth (Figure 2) [40].

Furthermore, NAC’s potent antioxidant activity has been found to interfere with insulin signaling by reducing insulin-induced H_2_O_2_ formation, which is required for proper insulin receptor activation, as NAC can contribute to insulin resistance [41].

## 3. RS and Endocrine Function

### 3.1. OS and RS as Pro-Pathogenic Conditions

The overexpression of antioxidant genes, excessive consumption of antioxidant food additives, and sustained overnutrition can lead to RS, which triggers ROS overproduction and OS [13,42]. The concept of OS has evolved significantly over time. Initially, OS was understood as an imbalance between free radical production and antioxidant defenses, leading to cellular and tissue damage [6]. However, more recent perspectives define OS as a general disruption of physiological redox signaling and redox regulation [43], emphasizing the role of redox-mediated signal transduction in maintaining cellular function [12]. This expanded definition of OS shifts its interpretation from merely a biochemical imbalance to a physiological state of distress—a condition of harmful influence and pathological processes. It contrasts with the broader concept of physiological stress, which is a universal adaptive response mobilizing body resources to cope with external stimuli. Physiological stress, including the well-known “fight or flight” response, is dominated by catabolic reactions and serves as an adaptive mechanism to counteract physical or psychological stressors (e.g., emotional distress) [43,44]. Conversely, acute or chronic stress can create vulnerable physiological conditions, contributing to the pathogenesis of various disorders and diseases [44]. Regardless of which definition of OS is adopted, the role of redox-mediated signal transduction in both OS and RS will continue to present complex challenges in redox biology [45,46].

ROS and RNS families comprise small, highly reactive molecules with short half-lives. Among them, membrane-permeable H_2_O_2_, with a half-life of a few seconds [47,48], and peroxynitrite (ONOO⁻), with a half-life of approximately one second [49], serve as key signaling mediators. To a lesser extent, superoxide anion, with a half-life in the millisecond range, plays a crucial role in mitochondrial signaling [50]. H_2_O_2_ is generated by over 30 different enzymes, making it the most important intracellular and paracrine redox messenger. While enzymatic systems efficiently regulate H_2_O_2_ levels, eukaryotic cells maintain nano-molar concentrations (1–100 nM) sufficient for the reversible oxidation of protein thiolate groups (R-SH), modulating protein function [51]. As a result, H_2_O_2_ influences numerous physiological processes, including Ca^2^⁺ influx [52], vascular smooth muscle contraction [53,54], angiogenesis, cell differentiation, migration, and apoptosis [47,52,55]. These adaptive physiological redox regulations, termed oxidative eustress, contrast with oxidative distress, which arises when supra-physiological H_2_O_2_ levels (>100 nM) contribute to inflammation, impaired vascular tone [53,54], fibrogenesis, tumor progression, metastasis, cell cycle arrest, and apoptosis [46,56,57].

Similarly, RNS play significant roles in both physiological and pathological conditions. While NO is well established as a signaling molecule in health and disease [58], peroxynitrite (ONOO⁻) acts as a potent oxidant and nitrating agent, modulating cellular pathways by nitrating tyrosine residues and interfering with intracellular processes regulated by tyrosine phosphorylation [59,60]. This leads to the formation of 3-nitrotyrosine-modified proteins in plasma, the vascular wall, and the cytoplasm [61], altering the function of ion channels, structural proteins, metabolic enzymes, growth factors, chemokines, and apoptosis-related proteins [59,60,61,62]. Additionally, peroxynitrite and other RNS can nitrate membrane lipids in human plasma and red blood cells, altering cell signaling and inducing DNA modifications. These modifications contribute to oxidative damage of membrane fatty acids and genomic DNA while triggering DNA repair mechanisms, further linking RNS to pathophysiological outcomes [60,63,64,65].

Overall, OS and RS act as interconnected pro-pathogenic conditions that disrupt redox homeostasis, leading to cellular dysfunction and disease progression. Their complex interplay underscores the need for precise redox regulation in maintaining physiological health.

### 3.2. RS, Diabetes, and Insulin Secretion

**Figure 3:**
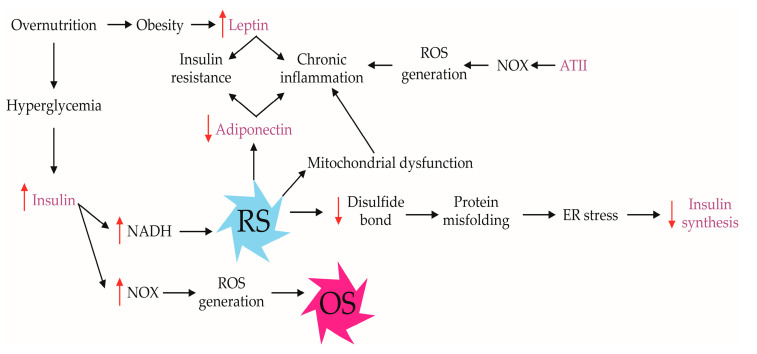
Metabolism-related endocrine regulations and RS. Chronic excessive nutrition associated with hyperglycemia and obesity disturbs endocrine regulation of metabolism accompanied by chronic inflammation. The condition is additionally complicated by the diversity of signaling pathways. (ATII: angiotensin II; NADH: nicotinamide adenine dinucleotide, reduced form; NOX: nicotinamide adenine dinucleotide phosphate oxidase; OS: oxidative stress; ROS: reactive oxygen species; RS: reductive stress). Black solid arrows (→) indicate direct cause-and-effect relationships or metabolic pathways. Bidirectional arrows (↔) represent reciprocal interactions or feedback loops. Red upward arrows (↑) indicate an increase or activation of specific molecules or processes. Red downward arrows (↓) indicate a decrease or inhibition of specific molecules or processes. Purple text indicates endocrine relationship with redox status.

The ER cisternae play a critical role in the proper modification, folding, and trafficking of secretory proteins, including polypeptide hormones [66]. Defective protein folding can be triggered by various factors, leading to ER stress, which is a predisposing factor for numerous human diseases [67]. In RS, excessive NADH causes mitochondrial dysfunction and protein misfolding in the ER, particularly by affecting disulfide bond formation, thereby triggering the unfolded protein response (Figure 3) [68].

Excessive expression of secretory proteins and genetically impaired N-linked glycosylation are among the primary causes of protein misfolding. ER stress, in turn, leads to reduced glucose-stimulated insulin secretion and hyperglycemia [69,70]. Consequently, the proper formation of secretory and membrane proteins, including insulin, is compromised due to impaired protein folding in the ER. Pancreatic β-cells are capable of producing approximately one million insulin molecules per minute [71]. However, ER stress-induced β-cell dysfunction is a key contributor to the pathogenesis of insulin-deficient diabetes (Table 1) [72]. It is widely accepted that OS contributes to β-cell dysfunction and insulin deficiency [73,74].

Hyperglycemia-induced OS is associated with a decline in the expression of endogenous antioxidant enzymes, such as peroxiredoxin 3 (a thioredoxin-dependent peroxide reductase), sirtuin 1, SOD, CAT, and GPx [75,76]. However, RS also impairs cellular function through distinct mechanisms [11]. RS primarily induces ER stress by disrupting the normally oxidizing environment within the ER, which is crucial for proper disulfide bond formation. This results in the loss of disulfide bonds during translation, compromising protein synthesis in both yeast [77] and human cells [78,79].

**Table 1:** RS in disease: principles and therapeutic implications.

Principles of RS-Mediated Disease	Therapeutic Implications	References
RS disrupts protein folding in the ER, leading to β-cell dysfunction and impaired insulin secretion.	Redox-modulating agents such as N-acetylcysteine (NAC) can help restore redox balance in metabolic diseases.	[66,67,79]
RS-induced inflammation exacerbates metabolic disorders by promoting macrophage polarization and cytokine secretion.	Targeting RS-associated inflammation through anti-inflammatory therapies may reduce metabolic complications.	[80,81]
RS influences metabolic hormones such as insulin, leptin, and adiponectin, as well as angiotensin II (AT II), impacting glucose homeostasis and immune responses.	Pharmacological interventions targeting NADPH oxidase (NOX) and redox-sensitive metabolic pathways could improve outcomes in RS-related diseases.	[82,83]
RS contributes to cardiovascular diseases by altering VSMC signaling, OS, and NO bioavailability.	Nanomedicine and targeted redox therapies show promise in selectively modulating RS for treating cardiovascular diseases and cancer.	[84,85]
RS affects epigenetic modifications, leading to changes in gene expression that influence disease progression.	Epigenetic therapies targeting RS-induced DNA methylation and histone modifications offer new avenues for treatment.	[86,87]
RS contributes to male infertility by disrupting sperm maturation, increasing oxidative vulnerability, and impairing DNA integrity.	Developing antioxidant formulations tailored for sperm function may help mitigate RS-induced infertility.	[88,89]

Insulin increases the NADH/NAD⁺ ratio and activates NAD(P)H oxidase (NOX) in cultured rat vascular (thoracic aorta) smooth muscle cells, thereby inducing RS and stimulating ROS production [90]. Similar effects were observed when NADH (1–3 µM) was applied to bovine heart mitochondria [91] or with the RS-inducing agent N-acetyl-L-cysteine (1 mM for 1 h) in rat L6 myoblasts [92]. Both treatments resulted in increased free radical leakage, suggesting that insulin, an elevated NADH/NAD⁺ ratio, and potentially other redox couples can promote RS-dependent free radical production, leading to OS. Notably, β-cells exhibit 2 to 3 times higher mitochondrial activity than other cell types, making them highly susceptible to redox imbalances [93].

Additionally, RS-induced hypoxia or elevated glucose levels can stimulate free radical formation through multiple mechanisms, including the inhibition of xanthine dehydrogenase (leading to increased superoxide production via xanthine/hypoxanthine oxidation), glycation-induced inhibition of CuZn-SOD, and NADH autoxidation, all of which contribute to cardiovascular dysfunction [94]. RS due to NADH accumulation in pancreatic β-cells can impair insulin secretion, primarily due to dysfunction of respiratory complex I and excessive free radical generation [95].

Sustained overnutrition-induced hyperglycemia and hyperlipidemia (glucolipotoxicity) can contribute to the development of diabetes mellitus via OS-sensitive mechanisms, which have been extensively reviewed elsewhere [96] and are not discussed here. Chronic overnutrition, particularly sustained fatty acid (FA) overload, is a major risk factor for type 2 diabetes, as it dysregulates the mechanistic target of rapamycin complex 1 (mTORC1) signaling, leading to impaired β-cell insulin secretion and reduced insulin sensitivity [97,98,99].

### 3.3. Metabolic Hormones, Angiotensin II (AT II), and Innate Immune Response in RS Conditions

Metabolic hormones, including insulin, leptin, and adiponectin, as well as AT II, play pivotal roles in regulating energy balance, glucose homeostasis, and immune responses (Figure 3). Under conditions of RS, the delicate redox balance within cells is disrupted, influencing both metabolic and immune pathways (Table 1).

Insulin modulates innate immunity by regulating macrophage polarization, promoting the anti-inflammatory M2 phenotype under physiological conditions but failing to counteract the pro-inflammatory M1 phenotype in chronic metabolic disorders [80].

AT II, a key regulator of blood pressure and of the monovalent cation homeostasis through aldosterone-dependent retention of sodium and excretion of potassium [100], is also influenced by RS. AT II stimulates NADPH oxidases (NOX) activity and increases ROS production, which further contributes to endothelial dysfunction and chronic inflammation. AT II inhibits insulin signaling in skeletal muscle cells through NOX-dependent and NOX-independent mechanisms [101]. The AT II–NOX axis is involved in the recruitment of monocytes and macrophages, amplifying the innate immune response and fostering a pro-inflammatory environment in metabolic tissues, such as adipose tissue, pancreas, and vascular endothelium (Figure 3) [81]. Additionally, AT II may impair insulin signaling through the phosphorylation of insulin receptor and insulin receptor substrate 1, which influences phosphoinositide 3-kinase (PI3K) activity downstream [102]. Therefore, AT II may regulate metabolism indirectly via insulin signaling, circulation, inflammation, and osmolarity, and, in some cases, this effect depends on RS.

Leptin, an adipokine predominantly secreted by adipocytes, plays a pivotal role in regulating energy balance and modulating immune responses. Under conditions of RS, leptin signaling can be disrupted, leading to metabolic and immune dysregulation. Elevated leptin levels, often observed in obesity, are associated with a pro-inflammatory state, as leptin promotes the activation and survival of various immune cells, including neutrophils and T lymphocytes. This heightened immune activation contributes to chronic inflammation and insulin resistance, exacerbating metabolic dysfunction in RS conditions (Figure 3) [82].

Adiponectin, another crucial adipokine, exhibits anti-inflammatory and insulin-sensitizing properties. Under RS conditions, adiponectin levels are often diminished, which correlates with increased OS and inflammation. Reduced adiponectin impairs its protective effects on metabolic tissues, leading to enhanced inflammatory responses and further promoting insulin resistance. The imbalance between leptin and adiponectin in RS states underscores the complex interplay between metabolic and immune pathways, highlighting the importance of maintaining redox homeostasis to preserve metabolic health (Figure 3) [83].

In obesity and diabetes, hypertrophic adipocytes secrete pro-inflammatory cytokines (e.g., TNF-α, IL-6, and MCP-1) and adipokines, disrupting metabolic-immune crosstalk. RS-induced mitochondrial dysfunction in adipocytes further enhances immune activation, leading to adipose tissue inflammation and exacerbating insulin resistance [103].

Overall, RS disrupts metabolic hormone signaling and triggers innate immune activation, creating a feedforward loop of chronic inflammation, redox imbalance, and metabolic dysfunction, particularly in insulin-resistant states such as diabetes, obesity, and cardiovascular disease.

### 3.4. RS as a Factor for Male Fertility

The full maturation of spermatozoa requires endogenous ROS production to mediate essential processes such as sperm hyperactivation, capacitation, and acrosomal reaction [104]. However, once in seminal fluid, sperm must be protected from oxidative damage by both enzymatic and non-enzymatic antioxidants, particularly SOD, CAT, and GPx [105,106].

RS can induce redox-sensitive transcription factors and gene expression changes, leading to developmental retardation and malformations [89]. In male gametes, NADH accumulation contributes to RS, which is associated with sperm motility defects and varicocele [107,108]. Dilated testicular veins impair the efficient drainage of blood from the testes, leading to venous stasis. This stagnation of blood increases local temperature and disrupts normal blood flow dynamics, ultimately reducing the delivery of oxygenated blood to the testicular tissue. This, in turn, activates HIF-1α signaling, leading to increased intracellular cysteine levels and GSH synthesis, which amplifies RS [5,109,110].

Under RS conditions, poly(ADP-ribose) polymerase (PARP) DNA repair activity is disrupted due to the depletion of NAD⁺ levels, resulting in an increased frequency of DNA damage [111]. Interestingly, antioxidants have also been identified as PARP inhibitors, as demonstrated in studies involving lung epithelial cells and ovarian cancer models [19].

Mature spermatozoa are particularly vulnerable to OS and RS because their DNA is highly condensed and transcriptionally inactive, preventing them from responding effectively to stress. Additionally, during sperm maturation, much of the cytoplasm, which serves as a primary source of antioxidants, is lost, further increasing their susceptibility to oxidative damage [112].

Overall, sperm maturation requires ROS; however, excessive RS disrupts motility, induces DNA damage, and increases oxidative vulnerability due to antioxidant depletion, emphasizing the importance of redox balance in male fertility competence.

### 3.5. Hormones That Suppress OS

Irisin, a myokine produced by skeletal muscle fibers during intense exercise, and the pineal gland hormone melatonin exhibit anti-inflammatory, antioxidant, neuroprotective, and anti-aging effects through both sirtuin 1-dependent and independent mechanisms [10,113,114]. Similarly, calcitriol (1,25-dihydroxyvitamin D_3_), the hormonally active metabolite of vitamin D, can also activate sirtuin 1 [114] and downregulate pro-inflammatory and growth signaling pathways, demonstrating anticancer activity in animal models of prostate and breast cancer [115]. These hormones induce a more reductive intracellular state; however, RS is not typically observed as a direct consequence of their secretion. In contrast, in individuals with obesity and possibly under other metabolic conditions, the supplementation of melatonin (2 mg/day) may have detrimental effects due to oscillations between OS and RS, disrupting redox homeostasis [116]. In this context, the result of melatonin supplementation resembles the pro-oxidant effects of antioxidants, as the ingestion of high doses of melatonin, similar to antioxidant vitamins, exerts direct pro-oxidant activity [116,117].

### 3.6. Challenges in RS Mediated Combat of Metabolic Disorders, Infertility, and Immune Complications

One of the primary challenges in targeting RS is the lack of precise biomarkers that can distinguish physiological redox states from pathological RS. While markers such as NADH/NAD⁺ and GSH levels serve as indicators, they are influenced by multiple metabolic processes. The development of sensitive and specific probes to quantify RS in real time remains a key area for future research.

The role of RS in diabetes and obesity is still not fully understood. While RS impairs insulin secretion and exacerbates inflammation, therapeutic strategies remain limited. A major challenge is the development of redox-modulating drugs that can selectively restore insulin sensitivity without disrupting normal metabolic function. Combining redox-targeted therapies with lifestyle modifications, such as exercise and dietary interventions, holds potential but requires further validation in clinical settings.

The effects of RS on male fertility present another challenge, as spermatozoa are highly susceptible to oxidative and RS. Future research should focus on targeting redox imbalances in infertility treatments, potentially by developing antioxidant formulations that support sperm function without exacerbating RS.

Another critical gap in knowledge is the interaction between RS and immune function. RS influences macrophage polarization, T-cell responses, and chronic inflammation, yet the exact mechanisms remain unclear. Identifying RS-dependent immune pathways could lead to novel immunomodulatory strategies for both autoimmune diseases and cancer immunotherapy.

To fully harness RS as a therapeutic target, future studies should aim to:

- Develop high-throughput screening assays for RS-targeting drugs;
- Investigate dietary and lifestyle interventions that modulate RS in metabolic diseases and aging;
- Explore personalized redox medicine, where genetic and metabolic profiling informs RS-targeted treatments;
- Establish clinical trials to evaluate RS-modulating therapies in diabetes and infertility.

Understanding the fine balance between OS and RS is essential for advancing therapeutic strategies that selectively exploit RS while maintaining cellular homeostasis. By integrating biochemical, metabolic, and clinical research, the field of redox medicine has the potential to redefine treatment paradigms across multiple diseases.

## 4. RS as a Factor for Cardiovascular Diseases and Cancer

**Figure 4:**
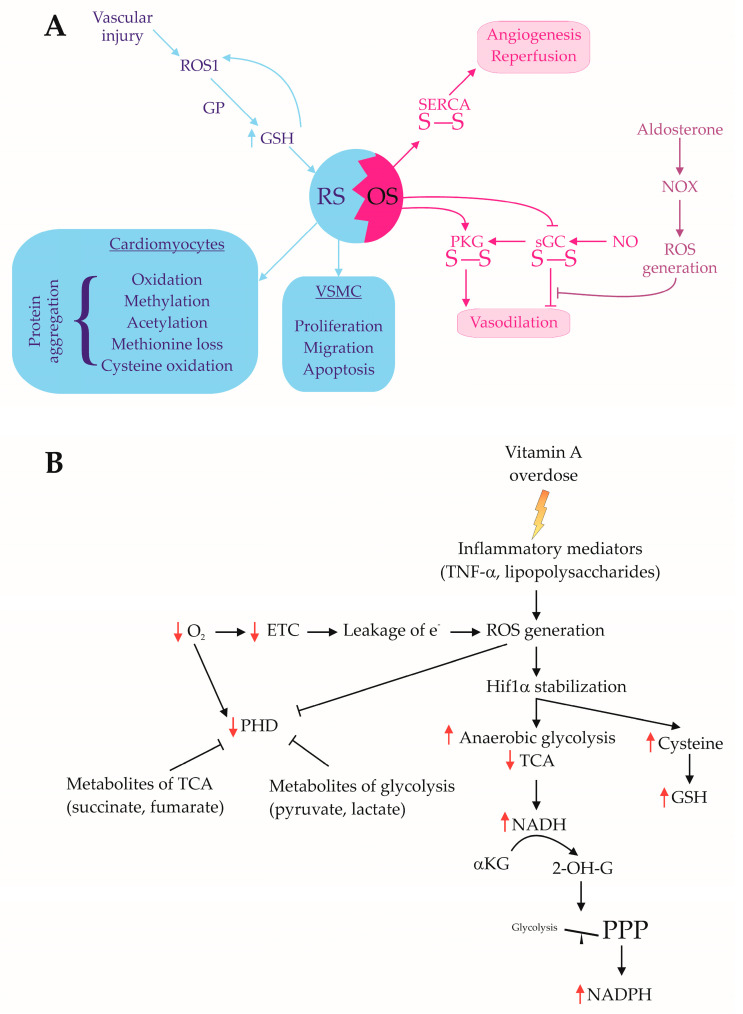
Hormone—RS cross regulations: (**A**) RS and cardiovascular changes. Increased concentration of reductive agents activates ROS1 intracellular resulting in increased GSH production which, in turn, keeps ROS1 in a phosphorylated, active state, forming a vicious circle. (**B**) Mechanism of hypoxia-induced RS. (2-OH-G; 2-hydroxyglutarate; αKG: alpha-ketoglutarate; ETC: electron transport chain; GP: glutathione peroxidase; GSH: glutathione, reduced form; NAD(P)H: nicotinamide adenine dinucleotide (phosphate), reduced form; NO: nitric oxide; NOX: nicotinamide adenine dinucleotide phosphate oxidase; OS: oxidative stress; PHD: oxygen sensor prolyl hydroxylase domain-containing protein; PKG: protein kinase G; PPP: pentose phosphate pathway; ROS: reactive oxygen species; proto-oncogene receptor tyrosine kinase ROS1; RS: reductive stress; sGC: soluble guanylate cyclase; TCA: tricarboxylic acid cycle; VSMC: vascular smooth muscle cells). A: Blue arrows (→) represent pathways involved in Reductive Stress (RS) and protective mechanisms. Pink arrows (→) indicate pathways associated with Oxidative Stress (OS) and its effects. Bidirectional interaction (↔) shows the balance and interaction between RS and OS, demonstrating how an imbalance in redox homeostasis can lead to cardiovascular dysfunction. B: Black solid arrows (→) represent direct metabolic or signaling events. Red upward arrows (↑) indicate increased production or activation of specific molecules or pathways. Red downward arrows (↓) indicate decreased activity or suppression of specific processes. Orange lightning bolt (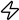) represents the influence of vitamin C overdose. Purple text indicates endocrine relationship with redox status.

Vascular injury activates the orphan proto-oncogene receptor tyrosine kinase ROS1 through endogenous phosphorylation. It also reduces GPx1 activity, leading to an elevated GSH level. GSH, in turn, inhibits protein tyrosine phosphatase SHP-2 through S-glutathiolation, thereby maintaining ROS1 in a phosphorylated and active state. Intracellular-activated ROS1, under conditions of RS, influences the proliferation, migration, and apoptosis of vascular smooth muscle cells (VSMCs) but not epithelial cells (Figure 4A) [118].

In transgenic mice with constitutive NRF2 activation, RS is induced, leading to alterations in the expression of more than 560 proteins (~50% of tested proteins), including 32 significantly changed proteins in cardiomyocytes. Post-translational modifications such as oxidation, methylation, acetylation, methionine loss, and cysteine oxidation were observed in NRF2-induced RS hearts, affecting both transcription and translation due to protein aggregation [119].

The redox status of VSMCs has paradoxical effects. Oxidation induces disulfide bond formation in protein kinase G (PKG), activating it independently of the NO/cGMP pathway. However, oxidant-induced disulfide bond formation in soluble guanylate cyclase (sGC) weakens the NO-mediated vasodilatory response. The NO/sGC/PKG pathway is impaired due to NADPH oxidase (NOX)-induced ROS production, which is driven by excessive aldosterone secretion [84]. In addition, SERCA (sarco/endoplasmic reticulum Ca^2^⁺-ATPase) is a redox-sensitive protein that is activated by disulfide bond formation, promoting angiogenesis and ischemic blood flow recovery (Figure 4A) [85].

Another unresolved contradiction remains: why are the detrimental effects of ROS overproduction not identical to those caused by antioxidant enzyme deficiencies? This discrepancy is partly explained by the compartmentalization of ROS and their specific subcellular distribution, affecting distinct cellular processes differently [120].

Hypoxia decreases mitochondrial ETC activity, leading to electron leakage and ROS generation. As a result, hypoxia-inducible factor-1α (HIF-1α) is stabilized, shifting metabolic energy production from oxidative phosphorylation to anaerobic glycolysis, which increases mitochondrial and cytosolic NADH levels [121]. Additionally, HIF-1α stabilization is influenced by TCA cycle metabolites (succinate, fumarate) and glycolytic intermediates (pyruvate, lactate) [122], as well as by inflammatory mediators such as TNF-α and lipopolysaccharides via ROS generation (Figure 4B) [123].

Hypoxia suppresses the expression of iron–sulfur cluster scaffold proteins, reducing the activity of mitochondrial ETC complexes I, II, and III, which contributes to NADH accumulation [124]. In response, hypoxic cells convert 2-ketoglutarate to 2-hydroxyglutarate using NADH, altering the glucose metabolism by shifting glycolysis towards the PPP. This adaptation reduces NADH production while increasing NADPH levels, providing an alternative redox balance mechanism [125]. Furthermore, the activation of the aspartate–pyruvate shuttle in hypoxic cardiomyocytes facilitates cytosolic NADPH production from mitochondrial NADH [126].

The accumulation of NADPH in cardiomyocytes during hypoxia serves as a protective role against ischemia-reperfusion injury, reducing oxidative damage upon oxygen restoration [127]. An understanding of the interaction between redox status and metabolism lays the basis for the development of therapeutic strategies to mitigate ischemia-reperfusion injury after myocardial infarction and improve cardiac outcomes during surgery.

Understanding the complex interplay between redox status and cardiovascular function remains a major challenge, as RS can both promote vascular dysfunction and protect against ischemia-reperfusion injury. Future research should focus on targeting redox imbalances in VSMC and cardiomyocytes, optimizing therapeutic strategies for myocardial infarction and ischemic heart disease, and clarifying the distinct effects of ROS compartmentalization on cardiovascular health.

### 4.1. Implications of RS on Cancer

While RS protects cancer cells from oxidative damage—enhancing survival and therapy resistance—it simultaneously disrupts redox signaling, impairing critical processes such as apoptosis and autophagy [128]. Dysregulated antioxidant systems further exacerbate this imbalance, fostering tumor progression. Paradoxically, excessive RS can also induce proteotoxic stress and metabolic dysfunction, highlighting its dual role in cancer pathophysiology [128]. Understanding these mechanisms may uncover redox vulnerabilities, paving the way for targeted cancer therapies.

#### 4.1.1. Pro-Tumorigenic Effects of RS

Reactive species play a crucial role in tumorigenesis by reprogramming cellular metabolism and promoting tumor growth through multiple interconnected mechanisms. One of the most well-characterized pathways involves its crosstalk with the Warburg effect, where cancer cells preferentially utilize aerobic glycolysis over oxidative phosphorylation. In this process, glutamine-derived α-ketoglutarate is converted into citrate, providing essential lipid biosynthesis precursors—a crucial requirement for the rapid proliferation of cancer cells [129,130,131]. The increased NADPH availability also plays a fundamental role in redox balance maintenance, as it facilitates the regeneration of GSH and Trx, both of which help neutralize ROS and maintain cellular homeostasis [132].

Beyond metabolic reprogramming, RS actively promotes tumor progression by stimulating angiogenesis and metastasis. This occurs, in part, through the upregulation of pro-angiogenic factors such as NO and vascular endothelial growth factor (VEGF), whose biosynthesis is enhanced by reductive metabolism [133,134]. Hypoxic conditions commonly found in the tumor microenvironment exacerbate these effects by stabilizing HIF-1α, a key regulator of VEGF expression and vascular remodeling [135]. This cascade promotes the formation of anomalous yet functionally efficient tumor vasculature, facilitating nutrient supply, immune evasion, and further tumor expansion.

In addition to promoting vascularization, RS contributes to tumor invasion and metastasis by modulating extracellular matrix (ECM) dynamics. A critical component of this process is the activation of matrix metalloproteinases (MMPs), a family of enzymes that degrade ECM components, allowing cancer cells to breach tissue barriers [136]. Elevated RS levels also enhance epithelial-to-mesenchymal transition (EMT), a process that increases migratory capacity and confers resistance to anoikis, a form of cell death triggered by detachment from the ECM. By sustaining these pro-metastatic adaptations, RS enables cancer cells to invade distant tissues and establish secondary tumors.

Furthermore, RS plays a crucial role in supporting the survival of metastatic cancer cells during their dissemination. Tumor cells circulating in the bloodstream are exposed to OS due to high ROS levels and immune surveillance. However, the RS-driven upregulation of antioxidant systems (such as elevated GSH and Trx activity) provides these cells with robust defense mechanisms, allowing them to resist oxidative challenges and successfully colonize distant organs [137].

This intricate interplay between metabolic adaptation, redox homeostasis, and invasive capacity underscores the multifaceted role of RS in driving cancer progression and therapy resistance. By further unraveling these mechanisms, novel therapeutic strategies targeting RS may be developed to improve cancer treatment outcomes.

#### 4.1.2. Anti-Tumorigenic Effects of RS

While RS plays a crucial role in supporting tumor progression, its excessive accumulation can paradoxically exert anti-tumorigenic effects by disrupting redox homeostasis and impairing ROS-mediated signaling pathways essential for cancer cell survival. ROS are fundamental in maintaining cellular redox balance and activating pro-survival pathways, including phosphatidylinositol 3-kinase/protein kinase B (PI3K/AKT) and mitogen-activated protein kinase (MAPK) signaling cascades. These pathways regulate cell proliferation, differentiation, and survival, enabling cancer cells to adapt to the tumor microenvironment. However, when RS levels become excessively high, they suppress ROS-dependent oncogenic signaling, compromising the activation of these key survival pathways.

A major consequence of this redox disruption is the destabilization of HIF-1α. Impaired HIF-1α stabilization leads to a reduction in VEGF expression, thereby limiting neovascularization and restricting the tumor’s ability to acquire nutrients and oxygen for sustained growth [138,139,140].

Beyond suppressing pro-tumorigenic signaling, excessive RS induces cellular damage by triggering proteotoxic stress and mitochondrial dysfunction, ultimately leading to apoptosis and senescence. The over-reduction of mitochondrial proteins disrupts ETC function, leading to energy depletion, metabolic collapse, and oxidative damage, culminating in cancer cell death [141]. Mitochondrial dysfunction further exacerbates OS by impairing ATP synthesis and increasing the leakage of pro-apoptotic factors such as cytochrome c, thereby activating caspase-dependent apoptotic pathways.

Additionally, RS enhances tumor suppression by stabilizing and activating tumor suppressor proteins, particularly p53, a key regulator of cell cycle arrest, DNA repair, and apoptosis. Excessive RS enhances p53 transcriptional activity, promoting the expression of p53 target genes that halt tumor progression and prevent malignant transformation [142]. The ability of RS to reinforce p53 function underscores its potential as a natural tumor-suppressive mechanism, which may be harnessed therapeutically to enhance the efficacy of existing anti-cancer treatments.

Interestingly, RS also disrupts autophagy, a survival mechanism that cancer cells exploit to withstand metabolic stress and therapy-induced damage. Under normal conditions, autophagy enables tumor cells to degrade damaged organelles and recycle essential biomolecules, ensuring metabolic flexibility. However, excessive RS impairs autophagic flux by disrupting lysosomal function and reducing autophagy-related protein expression, thereby making cancer cells more vulnerable to OS and cytotoxic agents [143]. This interference with autophagy represents a promising avenue for cancer therapy, as it may enhance tumor sensitivity to chemotherapy and radiation while reducing the likelihood of therapeutic resistance.

The dual-edged nature of RS highlights its complex role in cancer biology. While moderate RS levels promote tumor progression by supporting metabolic reprogramming and survival signaling, excessive RS can be leveraged therapeutically to drive OS beyond the tumor’s tolerance threshold. Exploiting this intrinsic redox vulnerability presents a promising strategy for selectively targeting cancer cells while sparing normal tissues. Future research into redox-based therapies may yield novel treatment approaches, enhancing cancer therapy efficacy and overcoming resistance mechanisms.

#### 4.1.3. Genetic Mutations in Redox Regulators and Their Impact on Cancer Progression

Genetic mutations in key redox regulators, such as tumor protein p53 (TP53) and kelch-like ECH-associated protein 1 (KEAP1), profoundly affect redox homeostasis, tumor progression, and therapeutic resistance.

TP53, a critical tumor suppressor gene, encodes the p53 protein, which regulates cell cycle arrest, DNA repair, apoptosis, and OS responses. Beyond these canonical functions, p53 plays a pivotal role in redox balance by modulating the expression of antioxidant genes, including GPx and sestrins [144,145,146]. However, TP53 mutations, commonly observed in various cancers, disrupt these regulatory mechanisms, leading to redox imbalances. Loss-of-function TP53 mutations can lead to elevated ROS levels, increasing genomic instability and promoting tumorigenesis. Gain-of-function TP53 mutations enhance reductive capacity, which contributes to chemoresistance and tumor survival by buffering ROS-induced damage [144]. Similarly, KEAP1, a key regulator of the NRF2 antioxidant pathway, plays a crucial role in maintaining redox homeostasis by promoting NRF2 degradation under normal conditions. However, KEAP1 mutations, frequently identified in lung adenocarcinoma, result in constitutive NRF2 activation, leading to upregulation of antioxidant enzymes. This persistent antioxidant response enhances cancer cell survival under OS, making tumors more resistant to therapy [147,148].

Moreover, co-mutations in KEAP1 and TP53 exacerbate these effects, promoting aggressive tumor phenotypes characterized by enhanced antioxidant defenses, metabolic reprogramming, and increased resistance to oxidative damage [149].

These genetic alterations underscore the interplay between redox regulation and tumor biology. Therapeutic strategies targeting TP53 and KEAP1 mutations, such as restoring wild-type p53 function or inhibiting NRF2 hyperactivation, hold promise for addressing redox imbalances and improving cancer treatment outcomes. Developing redox-based therapies tailored to specific genetic backgrounds may offer more effective interventions against therapy-resistant tumors.

#### 4.1.4. Impact of RS on the Epigenetic Landscape in Cancer

RS significantly influences the epigenetic landscape in cancer by modulating DNA methylation and histone modifications.

The redox environment directly affects the activity of DNA methyltransferases (DNMTs), which utilize S-adenosyl methionine (SAM) as a methyl donor for DNA methylation [75,150]. Excess-reducing equivalents, such as NADH and NADPH, can alter SAM availability and DNMT activity, leading to aberrant DNA methylation patterns. These changes may result in the silencing of tumor suppressor genes or the activation of oncogenes, ultimately promoting cancer progression [75]. Additionally, oxidative DNA damage, such as the accumulation of 8-oxo-2′-deoxyguanosine, interferes with normal methylation processes, further exacerbating epigenetic instability [151].

RS also modulates histone-modifying enzymes, including histone methyltransferases and histone acetyltransferases, which require specific redox conditions for optimal activity. Disruptions in redox balance can alter histone methylation and acetylation patterns, affecting chromatin structure and gene expression [95]. For example, aberrant histone methylation has been associated with the repression of tumor suppressor genes, while dysregulated histone acetylation can lead to impaired apoptosis and cell cycle control [87,152].

Understanding the interplay between redox states and epigenetic modifications provides a promising avenue for therapeutic intervention. Strategies targeting redox imbalances or epigenetic regulators may help reverse aberrant epigenetic marks, restore normal gene expression, and, ultimately, inhibit tumor progression [67].

### 4.2. Harnessing RS for Cancer Therapy: Challenges and Opportunities

**Figure 5:**
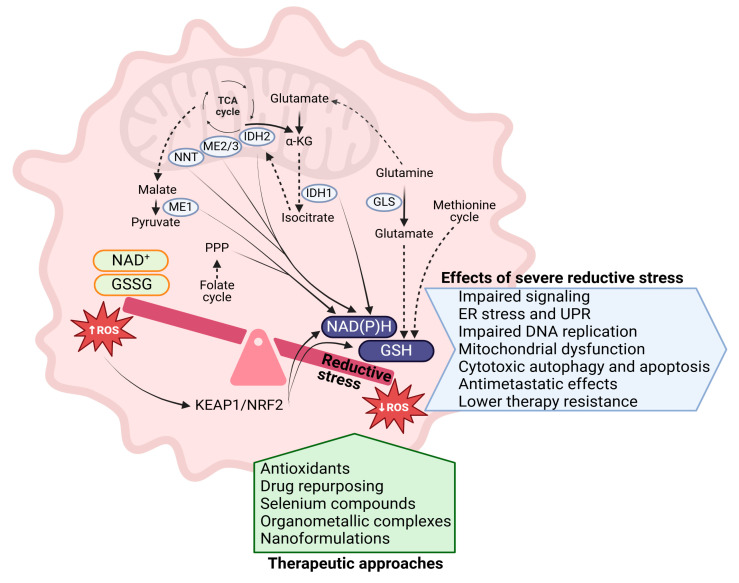
Exploiting RS in cancer. The figure illustrates key metabolic pathways contributing to the accumulation of NAD(P)H and GSH in cancer cells, resulting in a shift of the cellular redox state towards RS. While moderate levels of RS can support cancer cell survival and proliferation, excessive and sustained RS can be detrimental. It disrupts essential cellular processes, such as signaling pathways, protein folding, DNA replication, and mitochondrial function, leading to ER stress, mitochondrial dysfunction, and cytotoxic effects. The figure also highlights therapeutic approaches that have shown promise in further inducing RS to selectively target cancer cells. (GSH: glutathione, reduced form; GSSG: oxidized glutathione; KEAP1/NRF2: Kelch-like ECH-associated protein 1/nuclear factor erythroid 2-related factor 2; ME1/ME2/ME3: malic enzyme 1/2/3; NAD(P)H: nicotinamide adenine dinucleotide (phosphate), reduced form; NAD+: nicotinamide adenine dinucleotide, oxidized form; IDH1/2: isocitrate dehydrogenase 1/2; NNT: nicotinamide nucleotide transhydrogenase; GLS: glutaminase; PPP: pentose phosphate pathway; ROS: reactive oxygen species; α-KG: alpha-ketoglutarate; ER: endoplasmic reticulum; UPR: unfolded protein response; TCA cycle: tricarboxylic acid cycle). Solid arrows (→) indicate a direct biochemical reaction, conversion, or activation of one molecule into another. Dashed arrows (---→) indicate indirect interactions, regulatory pathways, or possible routes of influence. Bidirectional arrows (↔) represent reversible biochemical reactions or dynamic equilibrium between two states. Red arrows with "↑ROS" indicate an increase in ROS as a result of oxidative or reductive stress. Balancing scale suggests a regulatory balance between oxidative stress (↑ROS) and reductive stress (↓ROS), highlighting how the KEAP1/NRF2 pathway plays a role in maintaining homeostasis.

The intricate relationship between RS and cancer biology largely depends on NAD(P)H-generating pathways, with elevated NADH and GSH levels serving as key markers of RS (Figure 5). These insights have led to the exploration of therapeutic strategies aimed at inducing RS in cancer cells, demonstrating promising potential, even in treatment-resistant cancers.

Taking that RS may be leveraged against cancer, lifestyle interventions such as exercise-induced RS have been proposed as potential cancer-preventive strategies [153]. Additionally, significant attention has been directed toward modulating the cellular redox state through antioxidants as a therapeutic approach. Several studies support this concept. For example, patient-derived glioblastoma growth was inhibited in mice using N-acetylcysteine (NAC), a cysteine prodrug, which induced mitochondrial RS, leading to toxic levels of H_2_O_2_ production [154]. Similarly, under hypoxic conditions relevant to solid tumors, natural antioxidants such as resveratrol, curcumin, and celastrol were found to increase NAD(P)H levels, thereby inducing RS and leading to cell death in HepG2 cells [155].

Innovative redox-targeting strategies have also emerged, including biohydrogen-based approaches. To overcome challenges associated with molecular hydrogen delivery, probiotic biohydrogen microcapsules were developed to enable sustained hydrogen generation within the tumor microenvironment. This method effectively induced RS in multiple cancer cell lines, including drug-resistant models, and demonstrated efficacy in breast, melanoma, and liver cancer in vivo. The mechanism of action involved PI3K-AKT inhibition and MAPK activation, leading to cell cycle arrest and apoptosis [156]. However, caution is required when using antioxidants, as they may exhibit pro-oxidant properties at specific doses, potentially promoting cancer through mild RS induction. For instance, low ROS-lowering doses of vitamins C and A have been shown to accelerate malignant melanoma metastasis [157].

#### 4.2.1. Drug Repurposing and RS-Targeting Strategies

Beyond antioxidants, drug repurposing has been explored as a strategy for RS-mediated anticancer therapy (Table 2). For instance, carbidopa and the carbidopa–zinc complex have demonstrated efficacy against triple-negative human breast adenocarcinoma in vitro, selectively decreasing ROS levels, increasing GSH, and inducing mitochondrial damage and antimetastatic effects in MDA-MB-231 cells [158]. Additionally, selective RS induction and monitoring have been achieved through advanced molecular probes. A fluorescent probe specific for NAD(P)H enabled the real-time tracking of RS dynamics [156]. Furthermore, experiments using photoactivatable fluorescent probes that release trialkylphosphine, capable of reducing disulfide bonds and accumulating unfolded proteins, demonstrated the potential to induce and monitor RS in cancer cells [159].

#### 4.2.2. Selenium Compounds and Organometallic Complexes in RS Modulation

Several studies have highlighted the potential of selenium compounds and organometallic complexes in modulating cancer redox homeostasis (Table 2). Pharmacological doses of Na_2_SeO_3_ have been shown to induce RS, characterized by increased NADH and GSH levels, alongside an elevated reduced form of HMGB1. This process promoted autophagy-associated cell death via Akt/mTOR inhibition in HepG2 hepatocellular carcinoma cells [160]. Similarly, the redox-active organodiselenide 3,3′-diselenodipropionic acid effectively induced RS, ER stress, and p53-independent apoptosis in human lung cancer cells and in vivo models [161]. Likewise, 2,2′-dipyridyl diselenide (Py2Se2), a synthetic organodiselenide, was found to induce cell cycle arrest and apoptosis in non-small cell lung carcinoma [162].

Moreover, transition metal complexes have been utilized to manipulate RS in cancer therapy. Organo–ruthenium(II) complexes, when combined with formate as a hydride donor, effectively increased NADH levels in cancer cell lines [163,164]. Similarly, organo–rhodium(III) complexes facilitated hydride transfer under physiological conditions, effectively inducing RS in ovarian cancer cells [165]. More recently, platinum(II) N-heterocyclic carbene-based organometallic nucleosides have been found to selectively increase GSH levels and induce RS across multiple cancer cell lines [166].

#### 4.2.3. Nanomedicine and RS-Based Therapeutic Strategies

Nanotechnology has provided innovative delivery mechanisms for RS-inducing agents. pH-sensitive nanoformulations incorporating CdTe quantum dots and silica coatings were functionalized with poly(2-vinylpyridine)-polyethylene glycol-folic acid, enabling ascorbic acid delivery to induce RS and trigger apoptosis in hepatic cancer cells under hypoxia (Table 2) [167].

Other nanoformulations have demonstrated RS-inducing potential while improving drug delivery efficiency. For example, biocompatible magnetite (Fe_3_O_4_) nanoparticles coated with dextran or glucosamine-based amorphous carbon exhibited potent reductive activity. When tested in breast cancer cell lines, these nanoparticles reduced ROS levels while increasing antioxidant protein expression, cell cycle inhibitors, and autophagic markers, effectively inducing apoptotic cell death in chemotherapy-resistant senescent cancer cells [168].

Additionally, biomembrane-camouflaged nanomedicines containing polydopamine and ammonia borane have been developed to convert light into heat, triggering H_2_ release in the tumor microenvironment. This approach successfully eliminated tumors while reducing inflammation in a breast cancer model [55].

#### 4.2.4. Challenges and Future Directions

Despite the promising potential of RS-inducing therapies, resistance mechanisms may emerge. Cancer cells can sense and adapt to RS, shifting their redox balance through various mechanisms. For instance, the RS-induced binding of E3 ligase CUL2/FEM1B to mitochondrial FNIP1 leads to FNIP1 degradation, restoring mitochondrial ROS production and counteracting RS [169]. Furthermore, experimentally developed RS-resistant melanoma cells exhibited metabolic shifts, decreased ROS levels, and increased unfolded protein response activity, demonstrating that cancer cells can develop tolerance to both OS and RS [169].

However, recent advances suggest potential strategies to overcome RS resistance. For example, dimeric prodrug nanoassemblies with tetrasulfide (-SSSS-) bonds have been designed to selectively enhance RS in tumors while minimizing off-target toxicity [170]. Additionally, RS inducers can be combined with conventional chemotherapy to enhance cytotoxic effects, as seen with hydropersulfides (RSSH), which potentiate doxorubicin’s effects by inducing RS in various cancer cell lines [171].

## 5. Conclusions

RS has emerged as a crucial yet underexplored factor in metabolic and proliferative diseases, influencing diverse physiological processes through its interplay with key elements of redox homeostasis, endocrine regulation, and cellular metabolism. While OS has long been recognized as a driver of disease pathology, RS is increasingly implicated in conditions such as diabetes, obesity, cardiovascular disease, and cancer. The excessive accumulation of reducing equivalents, such as NADH and NADPH, disrupts cellular redox balance, altering insulin secretion, vascular function, and tumor progression. RS contributes to β-cell dysfunction and insulin resistance, promotes chronic inflammation in metabolic tissues, changes hormonal secretion and signaling, and enhances tumor survival by reinforcing antioxidant defenses. Excessive RS can also induce proteotoxic stress, mitochondrial dysfunction, and apoptosis, revealing a dual role in cancer biology.

Despite these insights, significant challenges remain in distinguishing physiological RS from pathological states and in developing targeted therapeutic interventions. Current strategies, including redox-modulating drugs, nanomedicine, and drug repurposing, offer promising avenues for selectively manipulating RS in disease contexts. However, therapy resistance and adaptive redox responses in cancer and metabolic disorders necessitate further research into the underlying molecular mechanisms. Future studies should focus on refining RS biomarkers, optimizing therapeutic strategies, and exploring the interaction between RS and immune function.

Understanding RS as a key factor of cellular homeostasis facilitates innovative treatments, integrating biochemical, metabolic, and clinical research approaches to advance redox equilibrium-based therapies for metabolic and proliferative diseases.

## Figures and Tables

**Table 2:** RS-related tumorigenic mechanisms and therapeutic implications.

Mechanism	Tumorigenic Effects	Therapeutic Implications	References
Warburg effect and NADPH overproduction	Cancer cells prefer aerobic glycolysis, leading to increased NADPH and glutamine-derived α-ketoglutarate conversion into citrate for lipid biosynthesis.	Targeting metabolic pathways (e.g., glycolysis and glutaminolysis) to disrupt RS-dependent tumor growth.	[129,130,131]
Angiogenesis and VEGF upregulation	RS enhances VEGF biosynthesis, promoting abnormal yet efficient tumor vasculature.	Anti-VEGF therapies and HIF-1α inhibitors to counteract RS-driven tumor vascularization.	[133,134,135,160]
EMT and metastasis	RS enhances MMP activation and EMT, facilitating invasion and metastasis.	MMP inhibitors and redox-modulating agents targeting EMT signaling.	[136,137]
Antioxidant system Upregulation	Elevated GSH and Trx levels protect tumor cells from OS and therapy-induced apoptosis.	GSH depletion strategies, Trx inhibitors, and redox-targeted chemotherapy.	[132]
RS-induced p53 activation	Excessive RS stabilizes p53, leading to tumor suppression via cell cycle arrest and apoptosis.	p53 reactivation therapies to exploit RS-induced cytotoxicity.	[142]
RS-triggered proteotoxic and ER stress	Excessive RS disrupts protein folding, induces proteotoxicity, and impairs autophagy.	Autophagy inhibitors and proteasome-targeted therapies to enhance RS-induced cell death.	[143]
Epigenetic modifications	RS alters DNA methylation and histone modifications, leading to gene expression dysregulation.	Epigenetic therapy targeting DNMTs and histone-modifying enzymes.	[74,87,150]
NRF2/KEAP1 mutations	KEAP1 mutations cause constitutive NRF2 activation, enhancing antioxidant defenses and therapy resistance.	NRF2 inhibitors and redox-targeted therapies to counteract therapy resistance.	[147,148,149]

## Data Availability

Not applicable.

## Abbreviations

The following abbreviations are used in this manuscript:

Akt	Protein kinase B
ALDH3A	Aldehyde dehydrogenase 3A
AMPK	AMP-activated protein kinase
AT II	Angiotensin II
CAT	Catalase
CuZn-SOD	Copper–zinc superoxide dismutase
DNMTs	DNA methyltransferases
ECM	Extracellular matrix
EMT	Epithelial-to-mesenchymal transition
ER	Endoplasmic reticulum
ETC	Electron transport chain
FNIP1	Folliculin-interacting protein 1
G6PD	Glucose-6-phosphate dehydrogenase
GSH	Glutathione
GPx	Glutathione peroxidase
HIF-1α	Hypoxia-inducible factor 1-alpha
HMGB1	High-mobility group box 1 protein
HMTs	Histone methyltransferases
KEAP1	Kelch-like ECH-associated protein 1
MAPK	Mitogen-activated protein kinase
ME1	Malic enzyme 1
MMPs	Matrix metalloproteinases
mTORC1	Mammalian target of rapamycin complex 1
NAC	N-acetylcysteine
NADH	Nicotinamide adenine dinucleotide (reduced form)
NADPH	Nicotinamide adenine dinucleotide phosphate (reduced form)
NF-κB	Nuclear factor kappa-light-chain-enhancer of activated B cells
NO	Nitric oxide
NOX	NADPH oxidase
NRF2	Nuclear factor erythroid 2-related factor 2
OXPHOS	Oxidative phosphorylation
OS	Oxidative stress
PARP	Poly(ADP-ribose) polymerase
PHD	Prolyl hydroxylase domain-containing protein
PI3K	Phosphoinositide 3-kinase
PKG	Protein kinase G
PPP	Pentose phosphate pathway
Py2Se2	2,2′-Dipyridyl diselenide
RA	Retinoic acid
RNS	Reactive nitrogen species
ROS	Reactive oxygen species
RS	Reductive stress
SAM	S-adenosyl methionine
SHP-2	Src homology region 2-containing protein tyrosine phosphatase
sGC	Soluble guanylate cyclase
SIRT1	Sirtuin 1
SOD	Superoxide dismutase
TCA cycle	Tricarboxylic acid cycle
TNF-α	Tumor necrosis factor alpha
TP53	Tumor protein p53
Trx	Thioredoxin
TSH	Thyroid-stimulating hormone
VEGF	Vascular endothelial growth factor
VSMCs	Vascular smooth muscle cells
8-oxo-dG	8-oxo-2′-deoxyguanosine
